# The nuclear receptor FXR inhibits Glucagon-Like Peptide-1 secretion in response to microbiota-derived Short-Chain Fatty Acids

**DOI:** 10.1038/s41598-019-56743-x

**Published:** 2020-01-13

**Authors:** Sarah Ducastel, Véronique Touche, Mohamed-Sami Trabelsi, Alexis Boulinguiez, Laura Butruille, Margaux Nawrot, Simon Peschard, Oscar Chávez-Talavera, Emilie Dorchies, Emmanuelle Vallez, Jean-Sébastien Annicotte, Steve Lancel, Olivier Briand, Kadiombo Bantubungi, Sandrine Caron, Laure B. Bindels, Nathalie M. Delzenne, Anne Tailleux, Bart Staels, Sophie Lestavel

**Affiliations:** 1 Université de Lille, Inserm, CHU Lille, Institut Pasteur de Lille, U1011- EGID, F-59000 Lille, France; 2 Université de Lille, CNRS, CHU Lille, Institut Pasteur de Lille, UMR 8199 - EGID, F-59000 Lille, France; 3 Metabolism and Nutrition Research Group, Louvain Drug Research Institute, Université Catholique de Louvain, 1200 Brussels, Belgium

**Keywords:** Endocrine system and metabolic diseases, Gastrointestinal hormones, Preclinical research, Nuclear receptors, Nutrient signalling

## Abstract

The gut microbiota participates in the control of energy homeostasis partly through fermentation of dietary fibers hence producing short-chain fatty acids (SCFAs), which in turn promote the secretion of the incretin Glucagon-Like Peptide-1 (GLP-1) by binding to the SCFA receptors FFAR2 and FFAR3 on enteroendocrine L-cells. We have previously shown that activation of the nuclear Farnesoid X Receptor (FXR) decreases the L-cell response to glucose. Here, we investigated whether FXR also regulates the SCFA-induced GLP-1 secretion. GLP-1 secretion in response to SCFAs was evaluated *ex vivo* in murine colonic biopsies and in colonoids of wild-type (WT) and FXR knock-out (KO) mice, *in vitro* in GLUTag and NCI-H716 L-cells activated with the synthetic FXR agonist GW4064 and *in vivo* in WT and FXR KO mice after prebiotic supplementation. SCFA-induced GLP-1 secretion was blunted in colonic biopsies from GW4064-treated mice and enhanced in FXR KO colonoids*. In vitro* FXR activation inhibited GLP-1 secretion in response to SCFAs and FFAR2 synthetic ligands, mainly by decreasing FFAR2 expression and downstream Gαq-signaling. FXR KO mice displayed elevated colonic FFAR2 mRNA levels and increased plasma GLP-1 levels upon local supply of SCFAs with prebiotic supplementation. Our results demonstrate that FXR activation decreases L-cell GLP-1 secretion in response to inulin-derived SCFA by reducing FFAR2 expression and signaling. Inactivation of intestinal FXR using bile acid sequestrants or synthetic antagonists in combination with prebiotic supplementation may be a promising therapeutic approach to boost the incretin axis in type 2 diabetes.

## Introduction

In addition to its major role in digestion and dietary nutrient absorption, the intestine also plays a role in glucose homeostasis through the interaction of nutrients and gut microbiota with enteroendocrine cells, hence modulating the secretion of numerous neuro-endocrine signals. Among these, the incretin Glucagon-Like Peptide-1 (GLP-1), which is synthetized as proglucagon precursor peptide, then cleaved and secreted by L-cells disseminated in a proximal to distal gradient in the gut epithelium^1^, exerts insulinotropic actions and controls glucose metabolism by binding to the GLP-1 receptors (GLP-1R) highly expressed on pancreatic beta cells^2^.

Enteroendocrine L-cells respond to various secretagogues through a range of sensing and signaling pathways including G-protein coupled receptors, ion channels and transporters^1^. Digested nutrients, such as glucose, amino acids and long-chain fatty acids, which are mostly absorbed in the small intestine, are GLP-1 secretagogues acting primarily in the small intestine^1,3^. By contrast, gut microbiota-derived metabolites, such as short-chain fatty acids (SCFAs), are mainly produced in the colon by fermentation of non-digestible polysaccharides. In addition to being important energy sources, SCFAs also act as signaling molecules and GLP-1 secretagogues by binding to the transmembrane receptors FFAR2 (GPR43) and FFAR3 (GPR41) to promote colonic L-cell response^4–8^. The bile acids (BAs), produced in the liver from cholesterol as primary BAs and transformed in the intestine into secondary BAs by the gut microbiota^9^ also modulate L-cell GLP-1 secretion by binding to the transmembrane receptor Takeda G-protein coupled Receptor 5 (TGR5)^10^ and the nuclear receptor Farnesoid X Receptor (FXR)^11,12^.

FXR, which is highly expressed in entero-hepatic organs (liver and intestine), plays a key role in the control of energy homeostasis by controlling BA, lipid^13–15^ and glucose metabolism^16–18^. Activation of hepatic FXR exerts beneficial effects on steatosis, inflammation, fibrosis and endothelial functions in the liver and could therefore be useful in the treatment of non-alcoholic steatohepatitis (NASH)^19^. Results from the FLINT trial showed that OCA (a steroidal FXR agonist) improves NASH and fibrosis in humans^20^. In line, many synthetic non-steroidal FXR agonists are under clinical development in humans for the treatment of NASH and fibrosis^19^. However, intestine-specific FXR-deficiency (^int^FXR KO) and pharmacological inhibition of intestinal FXR protect mice from high fat diet (HFD)-induced obesity, insulin resistance and non-alcoholic fatty liver diseases (NAFLD)^21–24^. Furthermore, treatment with BA sequestrants, anionic exchange resins trapping BAs in the intestinal lumen, hence increasing fecal BA excretion and de-activating intestinal FXR, improves lipid and glucose homeostasis by lowering LDL-cholesterol, decreasing intestinal glucose absorption, promoting splanchnic glucose utilization and increasing GLP-1 secretion^11,25–27^. De-activation of FXR in the intestine thus improves metabolic control. Given the role of insulin resistance and metabolic alterations in NAFLD, it is important to understand the role of FXR in the different metabolic organs to anticipate potential metabolic side effects in light of the clinical development of FXR agonists for NASH and fibrosis.

We previously showed that FXR is expressed in enteroendocrine L-cells where it regulates GLP-1 production in response to glucose^12^. FXR activation in L-cells decreases glucose-induced proglucagon gene expression and GLP-1 secretion through inhibition of the transcription factor ChREBP and the inhibition of glycolysis^12^. However, it is unknown whether FXR in L-cells also modulates the response to other secretagogues and especially the metabolites produced by the gut microbiota in the colon such as the SCFAs.

Therefore, GLP-1 secretion in response to SCFAs was evaluated *ex vivo* in intestinal biopsies from mice treated with GW4064, a synthetic FXR agonist, in murine WT and FXR KO colonoids and *in vitro* in murine and human L cells activated with GW4064. Expression of the SCFAs receptors FFAR2 and FFAR3 was also examined in these different models and FFAR2 Gαq-signaling pathway was evaluated *in vitro*. To assess *in vivo* the response to SCFAs, GLP-1 levels were measured in WT and FXR KO mice supplemented with prebiotics (inulin-type fructans) to increase SCFA production in the colon.

## Results

### FXR regulates GLP-1 secretion in response to SCFAs in the murine colon

**Figure 1:**
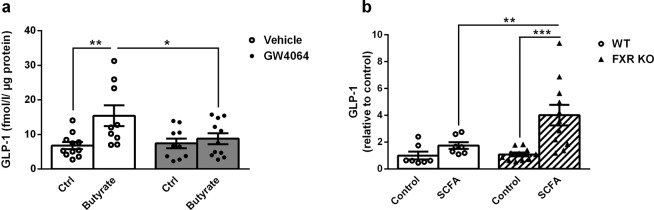
FXR regulates GLP-1 secretion in response to SCFAs *ex vivo* in the murine colon. (**a**) Active GLP-1 was measured in supernatants of colonic biopsies from WT mice 5 day-treated with vehicle or GW4064 (30 mg/kg), stimulated with control medium or medium plus Butyrate (1 mmol/l). Data are presented as mean ± SEM (white bars for vehicle-treated mice and grey bars for GW4064-treated mice). (n = 4 mice per group with 3 colonic biopsies per mouse and per stimulation condition). Two-way ANOVA followed by Bonferronni’s *post hoc* test. *p < 0.05 **p < 0.01. (**b**) Active GLP-1 was measured in supernatants of WT and FXR KO colonoids stimulated for 2 h with control buffer or buffer plus SCFA mix (acetate 5 mmol/l, propionate 1 mmol/l and butyrate 1 mmol/l). Fold induction compared to WT control condition which was set at 1 (absolute values (mean ± SD) of GLP-1 in the control condition: 0.07 ± 0.09 fmol/µg cell proteins). Data are presented as mean ± SEM of two independent experiments (white bars for WT colonoids and hatched bars for FXR KO colonoids). Two-way ANOVA followed by Bonferronni’s *post hoc* test. **p < 0.01 ***p < 0.001.

To assess whether FXR plays a role in the colonic L-cell response to SCFAs, an *ex vivo* GLP-1 secretion test in response to butyrate was performed on murine colon biopsies from WT mice treated orally for 5 days with vehicle or the synthetic FXR agonist GW4064 (30 mg/kg). *In vivo* GW4064 treatment activated colonic FXR as demonstrated by increased expression of FXR target genes such as *Fgf15* and *Ostβ*(data not shown). Butyrate induced GLP-1 secretion by colon explants from vehicle-treated mice by three-fold compared to control (Fig. 1a). This induction was totally blunted in explants from GW4064-treated mice (Fig. 1a). To assess the effect of FXR deficiency on the L-cell response to SCFAs, GLP-1 secretion experiments were performed on colonoids isolated from WT and FXR KO mice. While SCFAs (acetate 5 mmol/l, propionate 1 mmol/l, butyrate 1 mmol/l) slightly increased GLP-1 secretion in WT colonoids, the response to SCFAs was strongly increased in FXR KO colonoids (Fig. 1b). These results show that FXR regulates SCFA-induced GLP-1 secretion in the murine colon.

### FXR activation decreases GLP-1 secretion in response to SCFAs and synthetic FFAR2 agonists *in vitro* in murine and human L-cells

**Figure 2:**
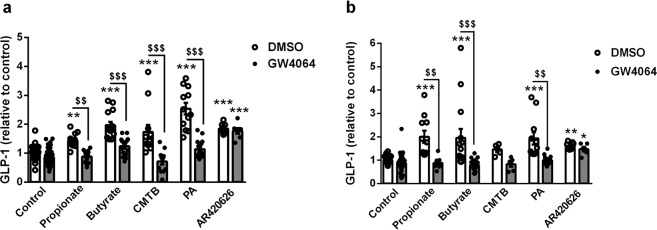
FXR activation decreases GLP-1 secretion in response to SCFA and synthetic FFAR2 agonists *in vitro* in murine and human L-cells. Active GLP-1 was measured in supernatants of murine GLUTag (**a**) and human NCI-H716 (**b**) cells treated for 24 h with GW4064 (5 µmol/l) and stimulated or not for 1 h (GLUTag) or 2 h (NCI-H716) with Glucose 5.6 mmol/l and Propionate 1 mmol/l, Butyrate 1 mmol/l, CMTB 10 µmol/l, PA 10 µmol/l or AR420626 10 µmol/l. Fold induction compared to control condition (DMSO treated cells/control medium) which was set at 1 (absolute values (mean ± SD) of GLP-1 in control conditions: DMSO treated GLUTag cells/control medium 0.88 ± 0.66 fmol/µg cell proteins; DMSO treated NCI-H716 cells/control medium 0.65 ± 0.51 fmol/µg cell proteins). Data are presented as mean ± SEM of at least three independent experiments (white bars for DMSO-treated cells and grey bars for GW4064-treated cells). Two-way ANOVA followed by Bonferronni’s *post hoc* test. *p < 0.05 **p < 0.01 ***p < 0.001 for secretagogue effect; ^$$^p < 0.01 ^$$$^p < 0.001 for FXR activation effect.

To determine whether the effect of FXR on the colonic response to SCFAs is L-cell intrinsic, the SCFA-induced GLP-1 secretion was examined in murine (GLUTag) and human (NCI-H716) L-cells^28,29^. Both GLUTag and NCI-H716 secreted GLP-1 in response to propionate and butyrate at 1 mM (Fig. 2a,b). As expected, FXR activation with GW4064 at 5 µmol/l for 24 h significantly increased FXR target gene expression such as *Fgf15* and *Ostβ*(data not shown). Interestingly, FXR activation also decreased propionate and butyrate-induced GLP-1 secretion in both human and murine cell lines (Fig. 2a,b).

Since SCFAs serve as energy sources for colonocytes^7^, we next explored whether L-cells metabolize butyrate and as such increase ATP levels and GLP-1 secretion. Incubation of GLUTag L-cells with butyrate significantly increased the ATP levels and basal respiration (increased Oxygen Consumption Rate) (Supplementary Fig. 1a,b), indicating that butyrate may also be used as energy source in L-cells. However, FXR activation did not modify these butyrate-induced cellular effects, as illustrated by the unchanged ATP and basal respiration rate upon GW4064 treatment (Supplementary Fig. 1a,b). Thus, FXR activation decreases SCFA-induced GLP-1 secretion without modifying SCFA metabolization in L-cells.

Since SCFAs are also signalling molecules and ligands of two different transmembrane G protein-coupled receptors, FFAR2 and FFAR3^30,31^, the impact of FXR activation on GLP-1 secretion in response to synthetic ligands of FFAR2 and FFAR3 was assessed. Different FFAR2 (4-CMTB and PA at 10 µmol/l) and FFAR3 (AR420626 10 µmol/l) synthetic agonists enhanced GLP-1 secretion by GLUTag and NCI-H716 cells (Fig. 2a,b). However, FXR activation decreased GLP-1 secretion only in response to 4-CMTB and PA, but not in response to AR420626, indicating that FXR regulation of SCFA-induced GLP-1 secretion occurs through FFAR2.

### FXR regulates *Ffar2*, but not *Ffar3* gene expression

**Figure 3:**
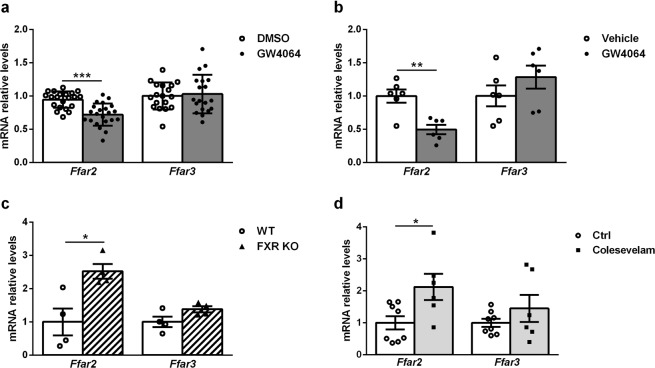
FXR regulates *Ffar2*, but not *Ffar3* gene expression. (**a**) *Ffar2* and *Ffar3* mRNA levels were quantified by qPCR on cDNA from GLUTag cells treated for 24 h with GW4064 (5 µmol/l). Data are presented as mean ± SD. Fold induction compared to control condition (DMSO) which was set at 1 (white bars for DMSO-treated cells and grey bars for GW4064-treated cells). Student’s t-test. ***p < 0.001. (**b**–**d**) *Ffar2* and *Ffar3* mRNA levels were quantified by qPCR on cDNA from colon of (**b**) WT mice treated by gavage for 5 days with vehicle or GW4064 (30 mpk) (n = 6 mice per group) (white bars for vehicle-treated mice and grey bars for GW4064-treated mice); (**c**) WT and FXR KO mice (n = 4 mice per group) (white bars for WT mice and hatched bars for FXR KO mice); (**d**) *ob/ob* WT mice treated with vehicle or Colesevelam (2%) for 3 weeks (n = 8 mice treated with vehicle and 6 mice treated with colesevelam) (white bars for control mice and light grey bars for colesevelam-treated mice). Data are presented as mean ± SEM. Student’s t-test *p < 0.05 **p < 0.01.

To assess whether the regulation of FFAR2-induced GLP-1 secretion by FXR may be due to regulation of SCFA-receptor gene expression, *Ffar2* and *Ffar3* mRNA levels were measured by RT-qPCR *in vitro* in GLUTag and *in vivo* in mouse colon. GW4064-treated GLUTag cells exhibited a decrease of *Ffar2* mRNA levels (Fig. 3a) in an FXR-dependent manner (Supplementary Fig. S2a). *Ffar2* mRNA levels were also significantly decreased in murine colons treated *in vivo* with GW4064 for 5 days (30 mg/kg) (Fig. 3b). On the contrary, *Ffar2* mRNA levels were increased in colons from FXR KO mice compared to their WT littermates (Fig. 3c) and in *ob/ob* mice treated for 2 weeks with the bile acid sequestrant colesevelam (Fig. 3d). By contrast, no significant changes in *Ffar3* mRNA levels were observed (Fig. 3a–d, Supplementary Fig. 2b). These results show that FXR inhibits *Ffar2* gene expression and suggest that FXR regulation of SCFA-induced GLP-1 secretion results, at least, in part from the downregulation of *Ffar2*, but not *Ffar3*, gene expression.

### FXR activation decreases FFAR2 Gαq-signaling in GLUTag L-cells

**Figure 4:**
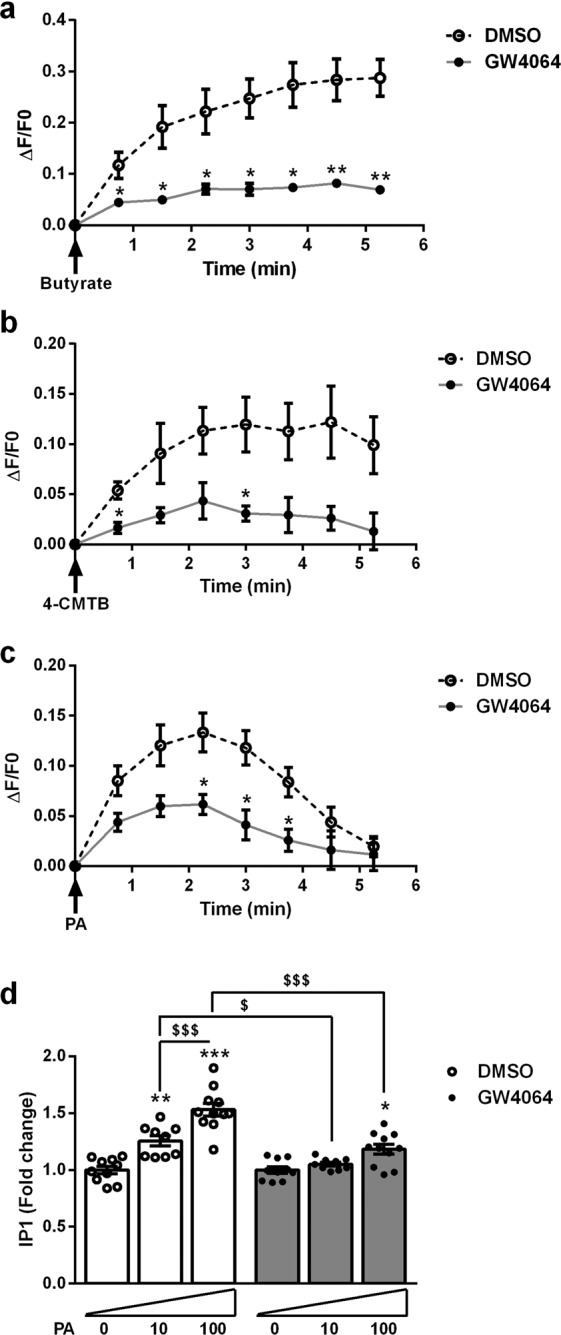
FXR activation decreases FFAR2 downstream Gαq-signaling in GLUTag L-cells. (**a**–**c**) GLUTag cells, treated or not with GW4064 5 µmol/l for 24 h, were loaded with Fluo-4AM. Calcium changes were assessed by calculating ΔF/F0 = (Fluorescence measured at each time point – Fluorescence at T0)/Fluorescence at T0, and normalized to fluorescence measured at each time point in non-stimulated control cells. Representative kinetic response to acute stimulation with butyrate (100 mmol/l) (**a**), 4-CMTB (10 µmol/l) (**b**), or PA (10 µmol/l) (**c**). Data are presented as mean ± SEM of three independent experiments. Student’s t-test *p < 0.05 **p < 0.01. (**d**) IP3 production was assessed with IP-One assay (Cisbio Bioassays) in GLUTag cells treated for 24 h with GW4064 (5 µmol/l) and stimulated or not for 90 min with PA 10 µmol/l or PA 100 µmol/l. Fold induction compared to non-stimulated cells which were set at 1 (absolute values (mean ± SD) of IP1 concentrations in control conditions: non-stimulated/DMSO cells 231.8 ± 76.8 nmol/L; non-stimulated/GW4064 cells 285.2 ± 89.1 nmol/L). Data are presented as mean ± SEM of three independent experiments (white bars for DMSO-treated cells and grey bars for GW4064-treated bars). Two-way ANOVA followed by Bonferronni’s *post hoc* test. *p < 0.05 **p < 0.01 ***p < 0.001 for significant difference to non-stimulated cells; ^$^p < 0.05 ^$$$^p < 0.001 for significant difference to other stimulated cells conditions.

To investigate whether FXR activation impacts on FFAR2-activated downstream Gαq-signaling^6^, the influence of FXR activation on the SCFA-induced Ca^2+^ response and inositol tri-phosphate (IP3) production (Gαq intracellular effectors) in GLUTag L-cells was evaluated. Acute stimulation with either butyrate, 4-CMTB or PA, increased intracellular Ca^2+^, an effect which was significantly lower in GW4064-pretreated cells (Fig. 4a–c). Inositol monophosphate (IP1) levels, reflecting IP3 production, dose-dependently increased in GLUTag cells upon stimulation with PA (Fig. 4d). By contrast, GW4064-pretreatment significantly attenuated the increase in IP1 level after stimulation with PA (Fig. 4d). Taken together these results show that the decrease in *Ffar2* gene expression upon FXR activation results in diminished downstream signaling in response to natural and synthetic agonists of FFAR2, an effect likely explaining the decrease in GLP-1 secretion.

### FXR deficiency enhances plasma GLP-1 levels in response to colonic SCFA supply in HFD-fed mice

**Figure 5:**
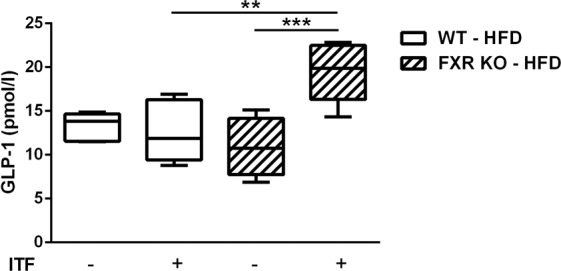
FXR deficiency enhances plasma GLP-1 levels in response to colonic SCFA supply in HFD-fed mice. Plasma levels of active GLP-1 were assessed, 1 h after Sitagliptin gavage, in FXR KO mice and their WT littermates after 14 weeks of HFD supplemented or not with the prebiotics ITF. Data are presented as boxplots (from the 25th to 75th percentiles). The whiskers are drawn down to the minimum and up to the maximum. (n = 6 mice per group, except in group FXR KO without ITF n = 4 mice). Two-way ANOVA followed by Bonferronni’s *post hoc* test. **p < 0.01 ***p < 0.001.

To assess whether inhibition of FXR could enhance SCFA-induced GLP-1 secretion *in vivo*, HFD-fed WT and FXR KO mice were supplemented with inulin-type fructans (ITF) (300 mg/mouse/day) which are known to increase the supply of microbiota-derived SCFAs to colonic L-cells and to improve energy homeostasis^32,33^. ITF-supplementation resulted in significantly higher active GLP-1 levels in plasma of FXR KO mice compared to WT mice (Fig. 5). Such increase in GLP-1 levels was also observed in chow diet-fed FXR KO mice supplemented with ITF (Supplementary Fig. 3a). Proglucagon (*Gcg*) and *Ffar2* mRNA levels were also higher in FXR KO mice compared to their WT littermates, but not affected by ITF supplementation (Supplementary Fig. 3b–d). *Ffar3* mRNA levels did not change under any of the tested conditions (Supplementary Fig. 3b–d).

## Discussion

The aim of the present study was to examine the role of the nuclear receptor FXR in the colonic L-cell response to gut microbiota-derived SCFAs. Consistent results demonstrating that FXR activation inhibits and FXR-deficiency potentiates GLP-1 secretion in response to SCFAs were obtained in different murine models (*in vivo*, colon biopsies, colonoids, *in vitro*). Interestingly, similar results were obtained in a human L-cell line suggesting that FXR may repress SCFA-induced GLP-1 secretion not only in mice, but also in humans. By regulating the SCFA-FFAR2 pathway, FXR may also modulate the secretion of other proglucagon-derived peptides, such as GLP-2, oxyntomodulin or glicentin. This interesting possibility warrants additional investigation. The decreased GLP-1 secretion response to SCFAs seems to involve a combination of both decreased expression of colonic Ffar2, but not Ffar3, and decreased FFAR2 downstream Gαq-related signaling. Moreover, FXR activation decreased GLP-1 secretion only in response to FFAR2 but not FFAR3 agonists, indicating that FXR activation specifically alters the SCFA-FFAR2 pathway regulating GLP-1 secretion. The molecular mechanisms behind the negative regulation of FFAR2 gene expression by FXR remain to be investigated.

Furthermore, we showed that plasma active GLP-1 levels increased in FXR-deficient mice after ITF supplementation, known to locally increase SCFA production. These results, consistent with our findings *in vitro* and *ex vivo*, correlated with an increase of both *Ffar2* and *Gcg* expression in FXR-deficient mice. Although several studies evidenced an increase in GLP-1-positive cells density in colon of rodents supplemented with ITF^34–37^, no significant increase in *Gcg* expression was found upon ITF supplementation in our study, indicating a signaling effect on existing L-cells.

Whether intestinal FXR activation or inhibition is metabolically beneficial remains under debate. *In vivo* treatment with fexaramine, an intestine-restricted FXR agonist, results in less weight gain, lower insulin resistance, decreased hepatic steatosis and increased beiging of adipose tissue in diet-induced obese (DIO) mice^38^, these beneficial effects being dependent on gut microbiota and BA/TGR5 signaling^39,40^. By contrast, numerous studies have shown that intestine-specific FXR-deficiency and pharmacological inhibition of intestinal FXR protect mice from HFD-induced obesity, insulin resistance and NAFLD^21–24^, these beneficial effects being mediated, at least in part, *via* decreased intestinal ceramide synthesis and secretion^41^. Furthermore, BA sequestrants, which de-activate intestinal FXR, induce metabolic improvements in animals models as well as in humans^11,12,25–27^. Again, there is evidence of a strong interaction between intestinal FXR and the gut microbiota. Comparing the metabolic phenotypes of conventionally-raised to germ-free mice under HFD, the intestinal microbiota promotes FXR-dependent weight gain and associated metabolic alterations such as hepatic steatosis and insulin resistance^42^. Mice treated with CAPE (caffeic acid phenethyl ester) an inhibitor of bacterial bile salt hydrolases (BSH)^43^, antibiotics^22^ or the antioxidant tempol^21,22^ display a change in BA pool composition, including an increase in tauro-β-muricholic acid, an FXR antagonist^44^, leading to an improved metabolic profile via intestinal inhibition of FXR. Furthermore a recent study suggests that the gut microbiota could participate to the metformin-induced metabolic ameliorations *via* re-shaping the microbiota towards bacteria producing BA FXR antagonists^45^. Our findings further confirm that FXR inhibition in the intestine *via* interaction with gut microbiota potentiates the metabolic control by enhancing the L-cell response to gut microbiota-derived SCFAs.

A better understanding of the mechanisms involved in the regulation of L-cell functions is crucial to identify new therapeutic targets to increase the incretin effect through regulation of both endogenous synthesis and secretion of GLP-1. As such, the nuclear receptors are interesting and targetable modulators of L-cell functions. We have previously shown that activation of the nuclear receptor PPARδ increases *Gcg* expression and glucose-induced GLP-1 secretion^46^ and that the nuclear receptor FXR inhibits *Gcg* expression and GLP-1 secretion in response to glucose in the small intestine^12^. In the present study, we focused on the colonic response since SCFAs are gut microbiota-derived metabolites produced in the colon^7^ and since the increase in GLP-1 and PYY secretion observed after bariatric surgery likely involves colonic L-cells^47,48^. Altogether, our findings identify FXR as an important regulator of L-cell functions all along the gut, regulating the L-cell incretin response to glucose in the ileum and to microbiota-derived metabolites in the colon. The combination of intestinal inhibition of FXR and prebiotic supplementation may constitute a promising approach in the management of metabolic disorders such as type 2 diabetes and their complications such as NAFLD and cardiovascular diseases. These data pave the way to test the combination of BA sequestration with inulin supplementation for the treatment of metabolic diseases in humans.

## Materials and Methods

Additional figures, protocols and methods details are described in the supplementary figures and methods section.

### Animals and *in vivo* protocols

Eight to twelve-week-old wild-type (WT) C57Bl6/J male mice (Charles River Laboratories, Wilmington, MA, USA) were gavaged for 5 days with 1% carboxymethylcellulose (CMC) containing or not GW4064 (30 mg/kg). Eight to ten-week-old male WT and FXR knock-out (KO) mice were fed a chow diet. Twelve to fifteen-week-old homozygous C57Bl6/J male WT mice on a leptin-deficient (*ob/ob*) background were fed *ad libitum* during 3 weeks with a standard diet (UAR A04, Villemoison/Orge, France) supplemented or not with 2% of colesevelam-HCl. Nine to twenty-week-old male FXR KO mice and their WT littermates were fed either chow diet or HFD (D12492; Research Diets; 60% kcal fat), and supplemented or not with inulin-type fructans (ITF) (Orafti P95, Beneo, Belgium) at 300 mg per mouse per day in the drinking water. For GLP-1 experiments, HFD-fed mice were 5h-fasted, then gavaged with the DPP-4 inhibitor Sitagliptin (25 mg/kg) (to allow plasma GLP-1 measurement) and 1 h later blood was collected by retro-orbital venipuncture under isoflurane anaesthesia. Chow diet-fed mice were gavaged with the long-acting DPP-4 inhibitor Omarigliptin (2 mg/kg), and blood was collected upon 7 h of fasting. All the experiments were approved and performed in accordance with the guidelines of Lille Pasteur Institute ethics committee (Agreement #2015121522544671) and EU Directive 2010/63/EU for animal experiments.

### *Ex vivo* and *in vitro* GLP-1 secretion assays

#### Murine intestinal biopsies

After a 30 min-glucose deprivation in glucose-free DMEM (Gibco), a 1h-GLP-1 secretion test was performed on colonic biopsies at 37 °C in DMEM supplemented with 100 µmol/l Diprotin A and 25 mmol/l glucose to which was added or not 1 mmol/l butyrate. Finally, medium was removed, centrifuged (1500 rpm, 5 min, 4 °C) to remove any floating cells or debris and immediately frozen at -80 °C. The remaining tissue was washed in cold PBS and frozen at -80 °C in NaOH 2 N. 3 colonic biopsies per mouse and per stimulation condition (control and butyrate) were used, from 4 mice per group (vehicle and GW4064). In parallel, GLP-1 secretion test was performed on 3 colonic biopsies per mouse in DMEM supplemented with 100 µmol/l Diprotin A and 25 mmol/l glucose to which was added 10 µmol/l FSK and 10 µmol/l IBMX as positive control of biopsy responsiveness.

#### Mouse colonoids

Three-day-differentiated colonoids in 48-well plates were washed 3 times then starved 30 min in glucose-free Hepes buffer consisting of 120 mmol/l NaCl, 5 mmol/l KCl, 2.2 mmol/l NaHCO_3_, 1 mmol/l MgCl_2_, 2 mmol/l CaCl_2_ and 10 mmol/l Hepes, supplemented with 0.1% BSA (pH = 7.2–7.4). Colonoids were subsequently incubated for 2 h with Hepes buffer supplemented with 100 µmol/l Diprotin A and 25 mmol/l glucose, to which was added or not a SCFA cocktail consisted of 5 mmol/l acetate, 1 mmol/l propionate and 1 mmol/l butyrate. Supernatants were collected, centrifuged (1500 rpm, 4 °C, 5 min), immediately frozen in liquid nitrogen and stored at -80 °C until analysis. Colonoids were washed in cold PBS, mechanically lysed in PBS, immediately frozen in liquid nitrogen and stored at -80 °C until analysis. Two independent experiments were performed, each in triplicates. Due to usual differences in GLP-1 basal secretion in separate colonoids experiments, all our results (active GLP-1 normalized to total protein content) were expressed as fold induction compared to WT control condition which was set at 1.

#### Cell lines

After 24 h-treatment in DMSO alone or supplemented with 5 µM GW4064^12^, GLUTag and NCI-H716 cells were starved for 30 min in glucose-free Krebs buffer consisting of 120 mmol/l NaCl, 5 mmol/l KCl, 2.2 mmol/l NaHCO_3_, 1 mmol/l MgCl_2_, 2 mmol/l CaCl_2_ and 20 mmol/l Hepes, supplemented with 0.2% bovine serum albumin (BSA) (pH = 7.2–7.4). Cells were then incubated for 1 h (GLUTag) or 2 h (NCI-H716) with Krebs buffer supplemented with 100 µmol/l Diprotin A and 5.6 mmol/l glucose, to which was added either 1 mmol/l propionate^6^, 1 mmol/l butyrate^6^, 10 µmol/l 4-CMTB^49^, 10 µmol/l PA^50^ or 10 µmol/l AR420626^50^. Supernatants were collected, centrifuged (1500 rpm, 4 °C, 5 min), transferred to fresh Eppendorf tubes and stored at −80 °C until analysis. Lysis samples were obtained by incubating cells with ice cold NaOH 0.4 M for 5 min while kept on ice. *In vitro* experiments were performed at least in triplicate and repeated at least 3 times.

#### GLP-1 measurements

Active GLP-1 in supernatants was measured with an enzyme-linked immunosorbent assay kit (EGLP-35K; Merck-Millipore) using Mithras Technology (Berthold) and normalized to the total quantity of cellular proteins. Protein contents were assessed by a BCA Protein Assay Kit (Pierce).

### Other *in vitro* studies

*In vitro* experiments were performed at least in triplicate and repeated at least 3 times.

#### Treatments and transient transfection assays

GLUTag and differentiated NCI-H716 cells were incubated for 24 h in DMEM supplemented with 0.2% BSA containing or not GW4064 (5 µmol/l). GLUTag cells were electroporated using the Neon Transfection System (Life Technologies) with small interference RNA against random (siCtrl) or Fxr (siFxr) sequences (smart pool sequences from Dharmacon Thermoscientific, see Supplemental Table 1). A total of 140,000 electroporated cells/cm^2^ seeded onto 24-well plates during 48 h were treated as described above.

#### Calcium flux assays

GLUTag cells were plated onto 96-well microplates 48 h before the study, then incubated for 24 h in DMEM supplemented with 0.2% BSA containing or not GW4064 (5 µmol/l). Cells were then loaded for 30 min with 2 µmol/l Fluo-4AM (cell-permeant formulation, Thermofisher) in Locke’s buffer consisted of 154 mmol/l NaCl, 4 mmol/l NaHCO_3_, 5 mmol/l KCl, 2,3 mmol/l CaCl_2_, 1 mmol/l MgCl_2_, 5 mmol/l glucose, 10 mmol/l Hepes. Cells were then washed and placed in Locke’s buffer. Fluorescence emission intensity in non-stimulated cells and cells acutely stimulated with Locke’s buffer containing 100 mmol/l butyrate or 10 µmol/l 4-CMTB or 10 µmol/l PA was measured using Infinite M200 Pro (Tecan) and Magellan software using the following settings: excitation 488 mm/emission 516 mm, Z position 17200 µm, readout every 45 sec, circle-type multiple reading (size 3 × 3, 750 µm border), temperature 37 °C. Calcium changes were assessed by calculating ΔF/F0 = (Fluorescence measured at each time point − Fluorescence at T0)/Fluorescence at T0, and normalized to fluorescence measured at each time point in non-stimulated control wells.

#### Evaluation of IP3 production by measurement of IP1 accumulation using homogeneous time-resolved FRET (HTRF)

Intracellular IP1 levels were measured using IP-One assay (Cisbio Bioassays, Codolet, France). GLUTag cells were plated onto 96-well white microplates 48 h before the study (80 000 cells per well), then incubated for 24 h in DMEM supplemented with 0.2% BSA containing or not GW4064 (5 µmol/l). Cells were washed with stimulation buffer (as supplied, including 50 mmol/l LiCl), then incubated for 90 min at 37 °C with 70 µl stimulation buffer containing or not the FFAR2 agonist PA as indicated. Cells were lysed by addition of 15 µl of the supplied buffer containing d2-labeled IP1, followed by 15 µl of buffer containing terbium cryptate-labeled anti-IP1 antibody, both reconstituted according to the manufacturer’s instructions. Plates were incubated for 1 h at room temperature and time-resolved fluorescence signals were measured at 620 and 665 nm using a Spectramax plate reader (Molecular Devices). HTRF ratio 665 nm/620 nm of each well were extrapolated on the standard curve to obtain IP1 concentrations. Fold change were then calculated based on these IP1 concentrations, with non-stimulated cells set at 1.

#### ATP measurements

ATP measurements on GLUTag cells in response to 1 mM butyrate were performed in the same culture conditions as GLP-1 secretion assays, using Cell Titer Glow assay (Promega) according to the manufacturer’s protocol. Luciferase activity was measured using Victor luminescence counter (PerkinElmer). Relative ATP levels were calculated from the measured luminescence setting the control condition (DMSO/control) at 1.

#### Analysis of oxygen consumption

Measurements of oxygen consumption rate (OCR) were performed using the XF24 analyzer (Seahorse Bioscience) on GLUTag cells seeded in XF24 V7 microplates for 48 h before a 24 h-treatment with 10 µmol/l GW4064. Basal OCR was measured in Seahorse assay buffer containing basic glucose-free DMEM medium (pH 7.4) supplemented or not with 1 mmol/l butyrate. Fold inductions were then calculated from these basal OCR measurements, with the control conditions (medium without butyrate) set at 1.

### Statistical analysis

Results are presented as mean ± SEM unless specified otherwise in figure legends. The experimental unit is single animal for *in vivo* experiments and is individual well for *ex vivo* and *in vitro* experiments. Statistical differences between two groups were assessed by unpaired Student’s t-test. Statistical differences between 3 or more groups were assessed by one-way or two-way analysis of variance (ANOVA) followed by Bonferronni’s *post hoc* test as indicated in figure legends. P values < 0.05 were considered significant. Statistical analyses were performed using GraphPad Prism version 7.0 for Windows.

## Supplementary information

Supplementary information.

## Data Availability

The data supporting this study are available from the corresponding author on reasonable request.

## References

[1] Reimann F, Gribble FM (2016). Mechanisms underlying glucose-dependent insulinotropic polypeptide and glucagon-like peptide-1 secretion. J. Diabetes Investig..

[2] Campbell JE, Drucker DJ (2013). Pharmacology, physiology, and mechanisms of incretin hormone action. Cell Metab..

[3] Psichas A, Reimann F, Gribble FM (2015). Gut chemosensing mechanisms. J. Clin. Invest..

[4] Gribble FM, Reimann F (2019). Function and mechanisms of enteroendocrine cells and gut hormones in metabolism. Nat. Rev. Endocrinol..

[5] Psichas A (2015). The short chain fatty acid propionate stimulates GLP-1 and PYY secretion via free fatty acid receptor 2 in rodents. Int. J. Obes. 2005.

[6] Tolhurst G (2012). Short-chain fatty acids stimulate glucagon-like peptide-1 secretion via the G-protein-coupled receptor FFAR2. Diabetes.

[7] Wichmann A (2013). Microbial modulation of energy availability in the colon regulates intestinal transit. Cell Host Microbe.

[8] Bindels LB, Dewulf EM, Delzenne NM (2013). GPR43/FFA2: physiopathological relevance and therapeutic prospects. Trends Pharmacol. Sci..

[9] Ridlon JM, Kang DJ, Hylemon PB, Bajaj JS (2014). Bile acids and the gut microbiome. Curr. Opin. Gastroenterol..

[10] Thomas C (2009). TGR5-mediated bile acid sensing controls glucose homeostasis. Cell Metab..

[11] Chávez-Talavera O, Tailleux A, Lefebvre P, Staels B (2017). Bile Acid Control of Metabolism and Inflammation in Obesity, Type 2 Diabetes, Dyslipidemia, and Nonalcoholic Fatty Liver Disease. Gastroenterology.

[12] Trabelsi M-S (2015). Farnesoid X receptor inhibits glucagon-like peptide-1 production by enteroendocrine L cells. Nat. Commun..

[13] Kuipers F, Bloks VW, Groen AK (2014). Beyond intestinal soap–bile acids in metabolic control. Nat. Rev. Endocrinol..

[14] Mazuy C, Helleboid A, Staels B, Lefebvre P (2015). Nuclear bile acid signaling through the farnesoid X. receptor. Cell. Mol. Life Sci. CMLS.

[15] Lefebvre P, Cariou B, Lien F, Kuipers F, Staels B (2009). Role of bile acids and bile acid receptors in metabolic regulation. Physiol. Rev..

[16] Caron S (2013). Farnesoid X receptor inhibits the transcriptional activity of carbohydrate response element binding protein in human hepatocytes. Mol. Cell. Biol..

[17] Prawitt J (2011). Farnesoid X receptor deficiency improves glucose homeostasis in mouse models of obesity. Diabetes.

[18] Cariou B (2006). The farnesoid X receptor modulates adiposity and peripheral insulin sensitivity in mice. J. Biol. Chem..

[19] Gege Christian, Hambruch Eva, Hambruch Nina, Kinzel Olaf, Kremoser Claus (2019). Nonsteroidal FXR Ligands: Current Status and Clinical Applications. Bile Acids and Their Receptors.

[20] Neuschwander-Tetri BA (2015). Farnesoid X nuclear receptor ligand obeticholic acid for non-cirrhotic, non-alcoholic steatohepatitis (FLINT): a multicentre, randomised, placebo-controlled trial. Lancet Lond. Engl..

[21] Li F (2013). Microbiome remodelling leads to inhibition of intestinal farnesoid X receptor signalling and decreased obesity. Nat. Commun..

[22] Jiang C (2015). Intestinal farnesoid X receptor signaling promotes nonalcoholic fatty liver disease. J. Clin. Invest..

[23] Zhang, L. *et al*. Farnesoid X Receptor Signaling Shapes the Gut Microbiota and Controls Hepatic Lipid Metabolism. *mSystems* **1** (2016). 10.1128/mSystems.00070-16 PMC5080402 27822554

[24] Jiang C (2015). Intestine-selective farnesoid X receptor inhibition improves obesity-related metabolic dysfunction. Nat. Commun..

[25] Prawitt J, Caron S, Staels B (2014). Glucose-lowering effects of intestinal bile acid sequestration through enhancement of splanchnic glucose utilization. Trends Endocrinol. Metab. TEM.

[26] Smushkin G (2013). The effect of a bile acid sequestrant on glucose metabolism in subjects with type 2 diabetes. Diabetes.

[27] McGettigan BM (2016). Sevelamer Improves Steatohepatitis, Inhibits Liver and Intestinal Farnesoid X Receptor (FXR), and Reverses Innate Immune Dysregulation in a Mouse Model of Non-alcoholic Fatty Liver Disease. J. Biol. Chem..

[28] Goldspink DA, Reimann F, Gribble FM (2018). Models and Tools for Studying Enteroendocrine Cells. Endocrinology.

[29] Kuhre RE (2016). Peptide production and secretion in GLUTag, NCI-H716, and STC-1 cells: a comparison to native L-cells. J. Mol. Endocrinol..

[30] Brown AJ (2003). The Orphan G protein-coupled receptors GPR41 and GPR43 are activated by propionate and other short chain carboxylic acids. J. Biol. Chem..

[31] Le Poul E (2003). Functional characterization of human receptors for short chain fatty acids and their role in polymorphonuclear cell activation. J. Biol. Chem..

[32] Roberfroid M (2010). Prebiotic effects: metabolic and health benefits. Br. J. Nutr..

[33] Cani PD, Delzenne NM (2011). The gut microbiome as therapeutic target. Pharmacol. Ther..

[34] Brooks L (2017). Fermentable carbohydrate stimulates FFAR2-dependent colonic PYY cell expansion to increase satiety. Mol. Metab..

[35] Cani PD, Hoste S, Guiot Y, Delzenne NM (2007). Dietary non-digestible carbohydrates promote L-cell differentiation in the proximal colon of rats. Br. J. Nutr..

[36] Everard A (2011). Responses of gut microbiota and glucose and lipid metabolism to prebiotics in genetic obese and diet-induced leptin-resistant mice. Diabetes.

[37] Catry E (2018). Targeting the gut microbiota with inulin-type fructans: preclinical demonstration of a novel approach in the management of endothelial dysfunction. Gut.

[38] Fang S (2015). Intestinal FXR agonism promotes adipose tissue browning and reduces obesity and insulin resistance. Nat. Med..

[39] Pathak P (2017). Farnesoid X receptor induces Takeda G-protein receptor 5 cross-talk to regulate bile acid synthesis and hepatic metabolism. J. Biol. Chem..

[40] Pathak P (2018). Intestine farnesoid X receptor agonist and the gut microbiota activate G-protein bile acid receptor-1 signaling to improve metabolism. Hepatol. Baltim. Md.

[41] Gonzalez FJ, Jiang C, Xie C, Patterson AD, Intestinal Farnesoid X (2017). Receptor Signaling Modulates Metabolic Disease. Dig. Dis. Basel Switz..

[42] Parséus A (2017). Microbiota-induced obesity requires farnesoid X receptor. Gut.

[43] Xie C (2017). An Intestinal Farnesoid X Receptor-Ceramide Signaling Axis Modulates Hepatic Gluconeogenesis in Mice. Diabetes.

[44] Sayin SI (2013). Gut microbiota regulates bile acid metabolism by reducing the levels of tauro-beta-muricholic acid, a naturally occurring FXR antagonist. Cell Metab..

[45] Sun L (2018). Gut microbiota and intestinal FXR mediate the clinical benefits of metformin. Nat. Med..

[46] Daoudi M (2011). PPARβ/δ activation induces enteroendocrine L cell GLP-1 production. Gastroenterology.

[47] Holst JJ (2013). Enteroendocrine secretion of gut hormones in diabetes, obesity and after bariatric surgery. Curr. Opin. Pharmacol..

[48] Larraufie P (2019). Important Role of the GLP-1 Axis for Glucose Homeostasis after Bariatric Surgery. Cell Rep..

[49] Bindels LB (2012). Gut microbiota-derived propionate reduces cancer cell proliferation in the liver. Br. J. Cancer.

[50] Christiansen CB (2018). The impact of short-chain fatty acids on GLP-1 and PYY secretion from the isolated perfused rat colon. Am. J. Physiol. Gastrointest. Liver Physiol..

