# Supplementary material for: The nuclear receptor FXR inhibits Glucagon-Like Peptide-1 secretion in response to microbiota-derived Short-Chain Fatty Acids
Source: Sci Rep. 2020 Jan 13;10:174. doi: 10.1038/s41598-019-56743-x (PMC6957696; doi:10.1038/s41598-019-56743-x)
Supplement: Supplementary file 1 — Supplementary information. [file 41598_2019_56743_MOESM1_ESM.pdf]

SUPPLEMENTARY FIGURES

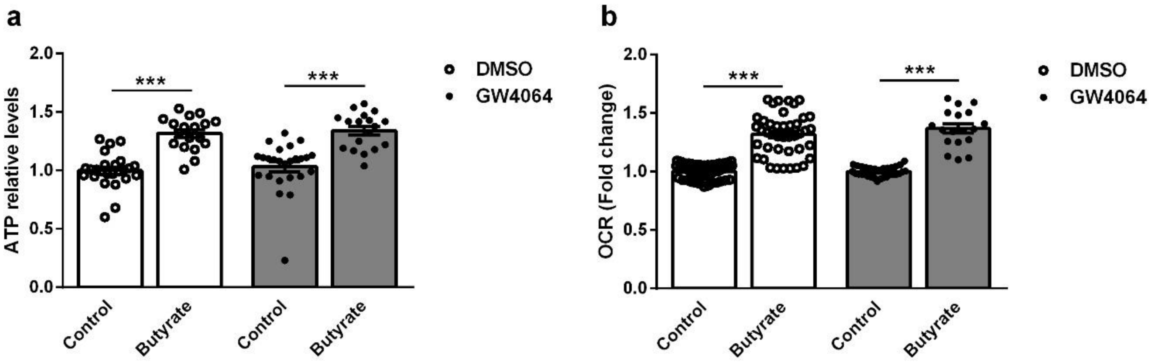

Supplementary Figure 1: FXR activation has no effect on Butyrate oxidation in GLUTag cells (**a**) Relative ATP levels were calculated from measured luminescence of GLUTag cells treated or not for 24h with GW4064 (5µmol/l) and stimulated or not for 1h with Butyrate (10mmol/l). Fold induction compared to control condition (DMSO/control) which was set to 1. (**b**) Basal respiration: Oxygen consumption rates were measured with Seahorse XF technology on GLUTag cells treated or not for 24h with GW4064 (5µmol/l) and then incubated in control medium supplemented or not with Butyrate (10mmol/l). Fold induction compared to control conditions (medium without butyrate) which were set at 1 (absolute values (mean±SD) in control conditions: DMSO/without butyrate 27.64±4.35 pmol/min/µg proteins; GW4064/without butyrate 32.47±5.71 pmol/min/µg proteins). Data are presented as mean±SEM (white bars for DMSO-treated cells and grey bars for GW4064-treated cells). Two-way ANOVA followed by Bonferronni's *post hoc* test. *** p<0.001.

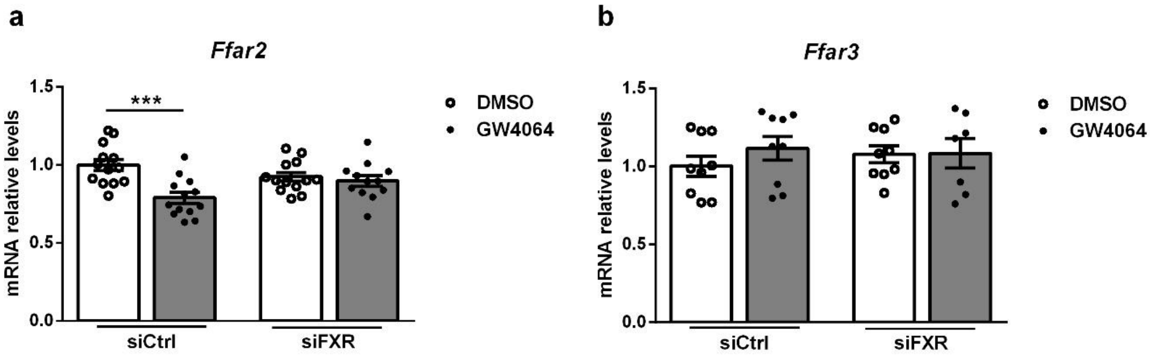

***Supplementary Figure 2: GW4064 decreases* Ffar2 *gene expression in an FXR-dependent manner, and not* Ffar3** (**a** and **b**) *Ffar2* and *Ffar3* mRNA levels were quantified by qPCR on cDNA from GLUTag cells electroporated with siCtrl or siFXR and treated for 24h with GW4064 (5µmol/l). Data are presented as mean±SD (white bars for DMSO-treated cells and grey bars for GW4064-treated cells). Fold induction compared to control condition (DMSO/siCtr) which was set at 1. Two-way ANOVA followed by Bonferronni's *post hoc* test. ***p<0.001.

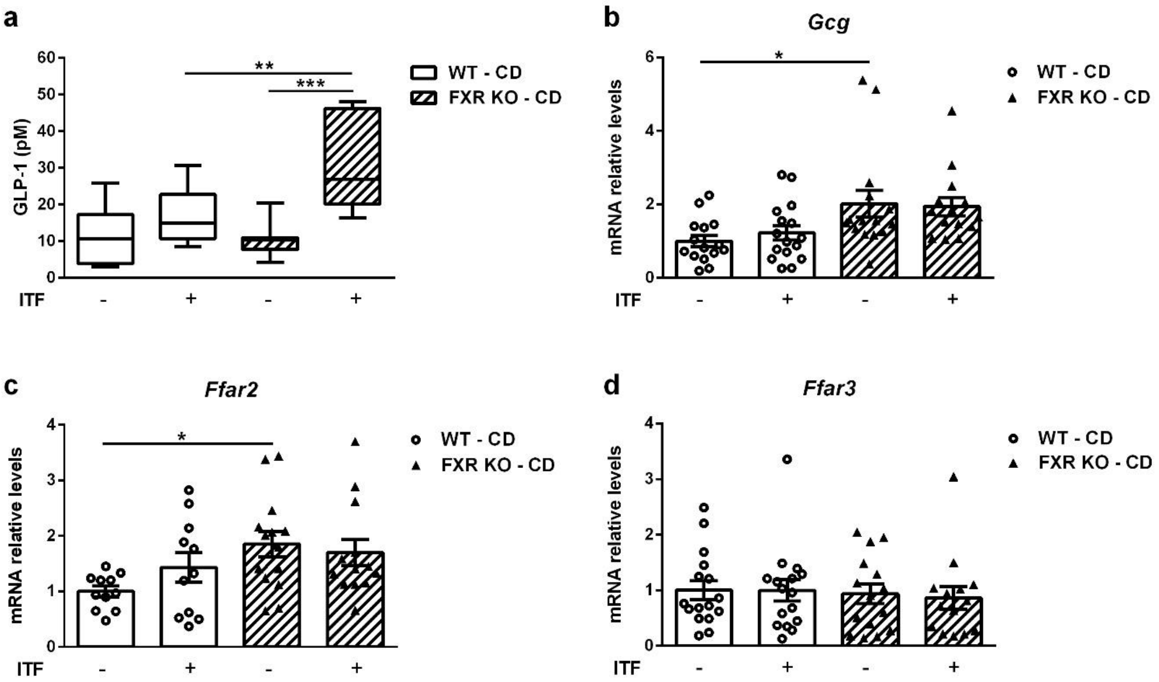

Supplementary Figure 3: Plasma GLP-1 levels are increased in chow diet-fed FXR KO mice supplemented with ITF (**a**) Plasma levels of active GLP-1 were assessed, 7h after Omarigliptin gavage, in FXR KO mice and their WT littermates on chow diet after 4 weeks of diet supplementation with the prebiotic ITF. Data are presented as boxplots (from the 25th to 75th percentiles) of one representative experiment with n=10 mice per group. The whiskers are drawn down to the minimum and up to the maximum. (white bars for WT mice and hatched bars for FXR KO mice) (**b to d**) mRNA levels of *Gcg* (**b**), *Ffar2* (**c**) and *Ffar3* (**c**) were quantified by qPCRs on cDNA from colon of FXR KO mice and their WT littermates after 6 weeks of the prebiotic ITF supplementation. Data are presented as mean±SEM of two independent experiments with respectively n=10 and n=7 mice per group (white bars for WT mice and hatched bars for FXR KO mice). Two-way ANOVA followed by Bonferronni's *post hoc* test. * p<0.05 *** p<0.001.

SUPPLEMENTARY MATERIALS AND METHODS

***Supplementary table 1**. Mouse small interfering RNA sequences*

Targeted gene	Dharmacon Smartpool sequences (5’→3’)
Fxr	GAAACUUCCUGCCGGACAU
	GUGUAAAUCUAAACGGCUA
	GAUUUGUGCCGGACGGGAU
	UGCCAGGAGUGCCGGCUAA

Chemicals and reagents

GW4064, Y-27632, DAPT and Wnt-C59 were purchased from Tocris (Bio-Techne, Lille, France). Short chain fatty acids (SCFAs, acetate, propionate, butyrate), the DPP-4 inhibitor diprotin A, the FFAR2 agonist 4-CMTB, the FFAR3 agonist AR420626, PD0325901, free fatty acid-bovine serum albumin (BSA), DMSO and CMC were purchased from Sigma-Aldrich (St Quentin-Fallavier, France). The FFAR2 agonist (S)-2-(4-chlorophenyl)-3,3-dimethyl-N-(5-phenylthiazol-2-yl)butanamide (PA) was purchased from Calbiochem (Merck Millipore, Burlington, Massachusetts, USA). GW4064, Y-27632, DAPT, Wnt-C59, 4-CMTB, PA, AR420626 and PD0325901 were dissolved in DMSO, Diprotin A and SCFAs were dissolved in high-purity water. Colesevelam-HCl was a kind gift of Daiichi Sankyo (Japan). Omarigliptine was purchased from MCE (Cat N° HY15981, MedChemExpress, Monmouth Junction, New Jersey, USA).

Animals and* in vivo *protocols

Mice in a C57Bl6/J background fed a chow diet were housed in a temperature-controlled room (22°C) on a 12h light–dark cycle, diet and water were *ad libitum.* In all experiments, male mice were naive of any king of procedure and randomized according to age and body weight. All the experiments were approved and performed in accordance with the guidelines of Lille Pasteur Institute ethics committee (Agreement #2015121522544671) and EU Directive 2010/63/EU for animal experiments.

Eight to twelve-week-old male wild-type (WT) mice (Charles River Laboratories, Wilmington, MA) were gavaged once a day in the morning for 5 days with 1% CMC containing or not GW4064 (30mg/kg) (n=6 mice per group, mean age at the end of protocol = 11 weeks, mean body weight = 27.6g for vehicle treated mice and 25.5g for GW4064 treated mice).

Eight to ten-week-old male WT and FXR knock-out (KO) mice were fed a chow diet (n=4 mice per group, mean age = 9 weeks, mean body weight = 26.3g for WT mice and 25.4g for FXR KO mice).

Twelve to fiveteen-week-old male WT mice on a leptin-deficient (*ob/ob*) background were fed *ad libitum* during 3 weeks with a standard diet (UAR A04, Villemoison/Orge, France) supplemented or not with 2% of colesevelam-HCl (n= 8 mice treated with vehicle, mean age = 16 weeks, mean body weight = 51.1g ; and n=6 mice treated with colesevelam, mean age = 17 weeks, mean body weight = 47g).

Nine to twenty-week-old male FXR KO mice and their WT littermates were separated in two groups, fed either chow diet or HFD (D12492; Research Diets; 60% kcal fat), and supplemented or not with inulin-type fructans (ITF) (Orafti P95, Beneo, Belgium) at 300mg per mouse per day in the drinking water. Body weight, food intake and water consumption were monitored weekly. Two independent experiments were performed under chow diet with respectively n=10 and n=7 mice per group. One experiment was performed under HFD with n=6 mice per group, except in the group of FXR KO mice without ITF n=4 mice (2 mice were excluded from this group due to injuries inflicted by their congeners associated with weight loss). Mean age and body weight are summarized in Supplementary table 2.

For GLP-1 experiments, HFD-fed mice were 5h-fasted, then gavaged with the DPP-4 inhibitor Sitagliptin (25mg/kg) (to allow plasma GLP-1 measurement) and 1h later blood was collected by retro-orbital venipuncture under isoflurane anaesthesia. Chow diet-fed mice were gavaged with the long-acting DPP-4 inhibitor Omarigliptin (2mg/kg) (to allow plasma GLP-1 measurement), and blood was collected by retro-orbital venipuncture under isoflurane anaesthesia upon 7h of fasting. Active GLP-1 in centrifuged plasma (10min at 4500rpm) was measured with a high-sensitivity enzyme-linked chemiluminescent assay kit (EZGLPHS-35K; Merck-Millipore) using Infinite M200 Pro (Tecan).

At the end of the protocols, after 5h of fasting, mice were killed by cervical dislocation. Colon were removed, washed once with phosphate-buffered saline (PBS), opened longitudinally on ice and the intestinal mucosa was scrapped and immediately frozen in liquid nitrogen. The samples were stored at -80°C for further analysis.

Supplementary table 2*. Age and body weight of mice in prebiotic supplementation protocols, on the day they were killed*

Experiment	Group	Mean Age (weeks)	Mean body weight (g)
HFD experiment	WT / HFD	34	44.4
	WT / HFD + ITF	34	45.3
	FXR KO / HFD	33	34.8
	FXR KO / HFD + ITF	33	31.4
Chow diet experiment N°1	WT	22	31.7
	WT + ITF	22	32.5
	FXR KO	23	24.9
	FXR KO + ITF	22	28.2
Chow diet experiment N°2	WT	17	28.2
	WT + ITF	17	28.7
	FXR KO	16	23.6
	FXR KO + ITF	16	20.2

In vitro and ex vivo cultures

Murine intestinal biopsies

Murine colonic biopsies from WT mice treated for 5 days with CMC or GW4064 (30 mg/kg) were isolated and processed as previously described [1]. Briefly, after 5h of fasting, mice were killed by cervical dislocation. The colon was placed in cold Hank's Balance Salt Solution (HBSS, Lonza) containing 2% horse serum (Life Technologies). Peyer's patches were removed, the colon was opened longitudinally and cut into 5-mm-long pieces. Pieces were washed 5 times in cold HBSS plus 2% horse serum and then incubated for 10min at 4°C in HBSS containing 2% horse serum and 1,4-dithiothreitol (DTT, 1mmol/l) to remove mucus. After additional washing in cold HBSS plus 2% horse serum, colon pieces were distributed in 24-well plates and stabilized for 3h at 37°C in Iscove's Modified Dulbecco Medium (Life Technologies) containing 10% fetal bovine serum (Life Technologies).

Crypt isolation and mouse colonoid culture

WT and FXR KO mice were killed by cervical dislocation. Colons were harvested and washed using syringe and needle with cold PBS. Colons were cut lengthwise then cut into 2mm pieces and rinsed several times in 50ml conical tubes in 15ml fresh cold PBS by vigorous shaking. Tissue pieces were

then resuspended in Gentle Cell Dissociation Reagent (Stemcell) and incubated at room temperature for 20min on a rocking platform. Tissue pieces were resuspended in PBS containing 0.1% BSA and up and down pipetted several times. After allowing pieces to settle by gravity, crypts were then collected by filter successively obtained supernatants through a 70µm filter and centrifuged at 290g for 5min at 4°C. The pelleted intestinal crypts were washed once in DMEM/F12 with 15mmol/l Hepes.

Five hundred crypts were seeded into Matrigel Matrix (Cat. No. 356231, Corning), in 48-well plates, in which they grow into colonoids, with Intesticult Organoid Growth Medium (Mouse) (Stemcell) and L-WRN cells-conditioned medium (L-WRN CM) (v/v), supplemented with 1% penicillin (10,000U/ml) / streptomycin (10,000µg/mL),10µM Y-27632 and then. L-WRN cell line (ATCC CRL-3276) was kindly provided by Benjamin Bertin (University of Lille, France) and L-WRN cells conditioned-medium was prepared as previously described [2]. The colonoid medium was refreshed every 2-3 days. Every week, colonoids were passaged by removing them from the Matrigel in Gentle Cell Dissociation Reagent (Stemcell), then pelleted, washed in DMEM/F12 and replated with a 1:3 splitting ratio in fresh Matrigel in 48-well plates.

The cocktail for enteroendocrine cell differentiation included: PD0325901 1µmol/l (MEK inhibitor), DAPT 10µmol/l (Notch inhibitor) and Wnt-C59 2µmol/l (Wnt inhibitor) [3,4] added to culture medium 3 days before GLP-1 secretion assay.

Cell lines

The mouse enteroendocrine L-cells GLUTag (mycoplasma-free, kindly provided by D.J. Drucker, University of Toronto, Toronto, Canada) were routinely grown at 37°C, 5% CO_2 in DMEM GlutaMAX 5.6mmol/l glucose (Cat. No. 21885-025, Gibco) supplemented with 10% fetal bovine serum (FBS), 1% penicillin (10,000U/ml) / streptomycin (10,000µg/ml) and split at 75% confluence.

The human enteroendocrine L-cells NCI-H716 (mycoplasma-free, ATCC® CCL-251™) were grown in suspension in RPMI 1640 medium (Cat. No. A10491-01, Gibco) supplemented with 10% fetal bovine serum (FBS), 1% penicillin (10,000 U/ml) / streptomycin (10,000µg/ml).

Forty-eight hours before experiments, GLUTag cells were plated onto 24-well plates and NCI-H716 cells were plated onto 24-well plates precoated with Matrigel® Basement Membrane Matrix (Corning) in DMEM (Cat. No. 41965, Gibco) supplemented with 10% fetal bovine serum (FBS), 1% penicillin (10,000 U/ml) / streptomycin (10,000µg/ml) to allow differentiation.

RNA extraction and quantification by qPCR

Total RNA were isolated from cells and tissues using Trizol reagent (Thermo Fisher Scientific) according to the manufacturer's protocol. After DNAse treatment (Thermo Fisher Scientific), cDNA were prepared by reverse transcription of 1µg total RNA using High capacity cDNA Reverse Transcription Kit (Applied Biosystems). Real-time qPCRs were performed with a Mx3000P instrument and MxPro software (Agilent Technologies) using the Brilliant II SYBR® Green QPCR Master Mix (Agilent). Sequences of primers are summarized in Supplemental Table 2. Results are presented using ddCt method normalized to cyclophilin (PPIA).

Supplementary table 3. *Primer sequences used for qPCR*

Gene	Species	Forward Primer	Reverse Primer
Ffar2	Mouse	5'-GAACGCTACCTGGGAGTGGC-3'	5'-ACGATGGTGCAGTGGCCAAA-3'
Ffar3	Mouse	5'-ACGTTTTCTGAGCGTGGCCT-3'	5'-AGCCGATGCCAGGAACCAAC-3'
Cyclophilin	Mouse	5'-GCATACGGGTCCTGGCATCTTGTCC-3'	5'-ATGGTGATCTTCTTGCTGGTCTTGC-3'
Gcg	Mouse	5'-GATCATTCCCAGCTTCCCAG-3'	5'-CTGGTAAAGGTCCCTTCAGC-3'

SUPPLEMENTAL REFERENCES

1. Trabelsi, M.-S. *et al.* Farnesoid X receptor inhibits glucagon-like peptide-1 production by enteroendocrine L cells. *Nat. Commun.* **6**, 7629 (2015).
2. Miyoshi, H. & Stappenbeck, T. S. In vitro expansion and genetic modification of gastrointestinal stem cells in spheroid culture. *Nat. Protoc.* **8**, 2471–2482 (2013).
3. Beumer, J. *et al.* Enteroendocrine cells switch hormone expression along the crypt-to-villus BMP signalling gradient. *Nat. Cell Biol.* **20**, 909–916 (2018).
4. Basak, O. *et al.* Induced Quiescence of Lgr5+ Stem Cells in Intestinal Organoids Enables Differentiation of Hormone-Producing Enteroendocrine Cells. *Cell Stem Cell* **20**, 177-190.e4 (2017).
